# Long non‐coding RNA as a novel biomarker and therapeutic target in aggressive B‐cell non‐Hodgkin lymphoma: A systematic review

**DOI:** 10.1111/jcmm.17795

**Published:** 2023-05-29

**Authors:** Shaghayegh Khanmohammadi, Parisa Fallahtafti

**Affiliations:** ^1^ Research Center for Immunodeficiencies, Pediatrics Center of Excellence, Children's Medical Center Tehran University of Medical Sciences Tehran Iran; ^2^ Non‐Communicable Diseases Research Center, Endocrinology and Metabolism Population Sciences Institute Tehran University of Medical Sciences Tehran Iran; ^3^ School of Medicine Tehran University of Medical Sciences Tehran Iran

**Keywords:** aggressive non‐Hodgkin lymphoma, biomarker, Burkitt's lymphoma, diffuse large B‐cell lymphoma, long non‐coding RNA, mantle cell lymphoma, non‐Hodgkin lymphomas

## Abstract

Cancer initiation and progression have been associated with dysregulated long non‐coding RNA (lncRNA) expression. However, the lncRNA expression profile in aggressive B‐cell non‐Hodgkin lymphoma (NHL) has not been comprehensively characterized. This systematic review aims to evaluate the role of lncRNAs as a biomarker to investigate their future potential in the diagnosis, real‐time measurement of response to therapy and prognosis in aggressive B‐cell NHL. We searched PubMed, Web of Science, Embase and Scopus databases using the keywords “long non‐coding RNA”, “Diffuse large B‐cell lymphoma”, “Burkitt's lymphoma” and “Mantle cell lymphoma”. We included studies on human subjects that measured the level of lncRNAs in samples from patients with aggressive B‐cell NHL. We screened 608 papers, and 51 papers were included. The most studied aggressive B‐cell NHL was diffuse large B‐cell lymphoma (DLBCL). At least 79 lncRNAs were involved in the pathogenesis of aggressive B‐cell NHL. Targeting lncRNAs could affect cell proliferation, viability, apoptosis, migration and invasion in aggressive B‐cell NHL cell lines. Dysregulation of lncRNAs had prognostic (e.g. overall survival) and diagnostic values in patients with DLBCL, Burkitt's lymphoma (BL), or mantle cell lymphoma (MCL). Furthermore, dysregulation of lncRNAs was associated with response to treatments, such as CHOP‐like chemotherapy regimens, in these patients. LncRNAs could be promising biomarkers for the diagnosis, prognosis and response to therapy in patients with aggressive B‐cell NHL. Additionally, lncRNAs could be potential therapeutic targets for patients with aggressive B‐cell NHL like DLBCL, MCL or BL.

## 1 INTRODUCTION

Non‐Hodgkin lymphoma (NHL) encompasses a group of heterogeneous malignancies of lymphoid cells originating from mature or immature B lymphocytes, T lymphocytes or natural killer (NK) cells.^1^ The global age‐standardized incidence rate of NHL in 2020, the most common haematologic malignancy, was 5.8 per 100,000 persons. The increasing trend of NHL incidence and mortality rates puts NHL as an important cause of global cancer burden.^2^ Although there is geographical variation in the prevalence of NHL, it remains a major cause of morbidity in most regions.

Based on the prognosis of the disease, NHL is classified into two groups: indolent and aggressive NHL. Follicular lymphoma, chronic lymphocytic leukaemia, splenic marginal zone lymphoma and small lymphocytic lymphoma usually present with indolent manifestations, while diffuse large B‐cell lymphoma (DLBCL), B‐cell and T‐cell lymphoblastic leukaemia/lymphoma, Burkitt's lymphoma (BL), mantle cell lymphoma (MCL) and adult T‐cell leukaemia/lymphoma are known as aggressive NHL.^1^

Over the years, the role of genetics, epigenetics and molecular alternations has been revealed in the pathogenesis of NHL.^3^ Abnormalities in karyotype and mutations contribute to the development of NHL. Despite improvements in chemotherapy‐based treatments, the outcome of aggressive NHL has remained poor.^4^ The heterogeneity of NHL subtypes and differences in their presentations, prognosis and response to treatment highlight the need for more specific diagnostic, prognostic and therapeutic biomarkers.

Long non‐coding RNAs (lncRNAs) are a group of non‐coding RNAs consisting of more than 200 nucleotides transcribed from intergenic, genic and enhancer regions.^5^ LncRNAs play a crucial role in modulating gene expression at different epigenetic, transcriptional, post‐transcriptional, translational and post‐translational levels.^6^, ^7^ Alternations in lncRNAs may lead to changes in the cell cycle, transforming normal cells into malignant cells. Recently, many studies have demonstrated that lncRNAs are involved in different malignancies, including lymphomas. In haematologic malignancies, lncRNAs may affect cell proliferation, invasion and resistance to treatment through different pathways.^8^

Furthermore, studies have shown changes in lncRNA expression in different stages of the maturation of lymphocytes.^9^ Understanding the lncRNA mechanism of action provides more information about lymphoma's pathogenesis. Additionally, it can explain the drug‐resistant disease and may improve treatment outcomes. In recent years, increasing studies on B‐cell NHL have been conducted to determine the expression profile of lncRNAs. For example, MALAT1 lncRNA is overexpressed in DLBCL cell lines resistant to chemotherapy, inhibiting autophagy pathways.^10^ NONHSAG026900 lncRNA is another highly expressed lncRNA in DLBCL patients with a more favourable outcome. DLBCL patients with high levels of NONHSAG026900 are more responsive to chemotherapy than patients with lower levels.^11^ More recent evidence focuses on lncRNAs as potential diagnostic and prognostic disease biomarkers and therapeutic targets, as they are tissue‐specific markers detected easily in body fluids.^12^, ^13^

In this systematic review, we evaluated the studies on the role of lncRNAs in aggressive B‐cell NHL as a biomarker to investigate their future potential in diagnosis, real‐time measurement of response to therapy and prognosis.

## 2 MATERIALS AND METHODS

This study followed the guidelines in the Preferred Reporting Items for Systematic Reviews and Meta‐Analyses (PRISMA) Statement.^14^ The protocol of our systematic review was registered at PROSPERO with the registration number CRD42022358768.

### 2.1 Literature search

We comprehensively searched the electronic databases PubMed, Web of Science (ISI), SCOPUS, and EMBASE for original articles from inception to 4 March 2023. The search strategy was built in PubMed, and subsequently, databases were searched through the following medical subject headings (MeSH) terms and free keywords: “long non‐coding RNA”, “Diffuse large B‐cell lymphoma”, “Burkitt's lymphoma”, “Mantle cell lymphoma” and their expansions. The search strategy is available in the Supporting Information. All records were transferred to EndNote software 20, and duplicates were removed.

### 2.2 Selection criteria

In the final analysis, we only included the papers that met all the following criteria: (1) original studies; (2) studies reporting on human subjects with aggressive B‐cell NHLs; (3) studies with control groups; (4) studies measuring lncRNA levels; (5) English‐language studies; (6) full‐text available.

We excluded book chapters, commentary articles, case reports, letters, editorials, review articles and conference abstracts. Studies with human samples from gene datasets were also excluded from this study.

Two authors (SK and PF) screened titles and abstracts of all identified studies for eligibility based on predefined inclusion and exclusion criteria. After collecting eligible studies, two authors (SK and PF) independently conducted a comprehensive full‐text review. Conflicts were resolved through consensus.

### 2.3 Data extraction

Two reviewers (SK and PF) extracted data from eligible studies in a dedicated electronic spreadsheet. Conflicts were resolved through consensus. For each included study, the following data were extracted when available: author name, publication year, study method, specimen type, sample size, lncRNA name, levels of lncRNA in patients with lymphoma compared to the control group, marker type, the role of lncRNA in lymphoma pathogenesis, main findings and measures of effect (if available).

### 2.4 Quality assessment

Two authors (SK and PF) independently assessed the quality of included studies using the Newcastle‐Ottawa Quality Assessment Scale (NOS) for observational studies^15^ to determine the risk of bias in the included studies. Any discrepancies were resolved through discussion between the authors. The NOS has three main categories of bias: selection, comparability and outcome. Scores of 7–9, 4–6 and 0–3 considered ‘good’, ‘fair’ and ‘poor’ studies in terms of quality, respectively.

### 2.5 Statistical analysis

Due to expected heterogeneity among included studies in terms of study design, type of aggressive B‐cell NHLs and lncRNA, control group and methods used for lncRNA detection, we planned to conduct a narrative synthesis rather than a meta‐analysis. Descriptive statistics were used in Microsoft Excel 2016.

## 3 RESULTS

### 3.1 Study characteristics

**FIGURE 1:**
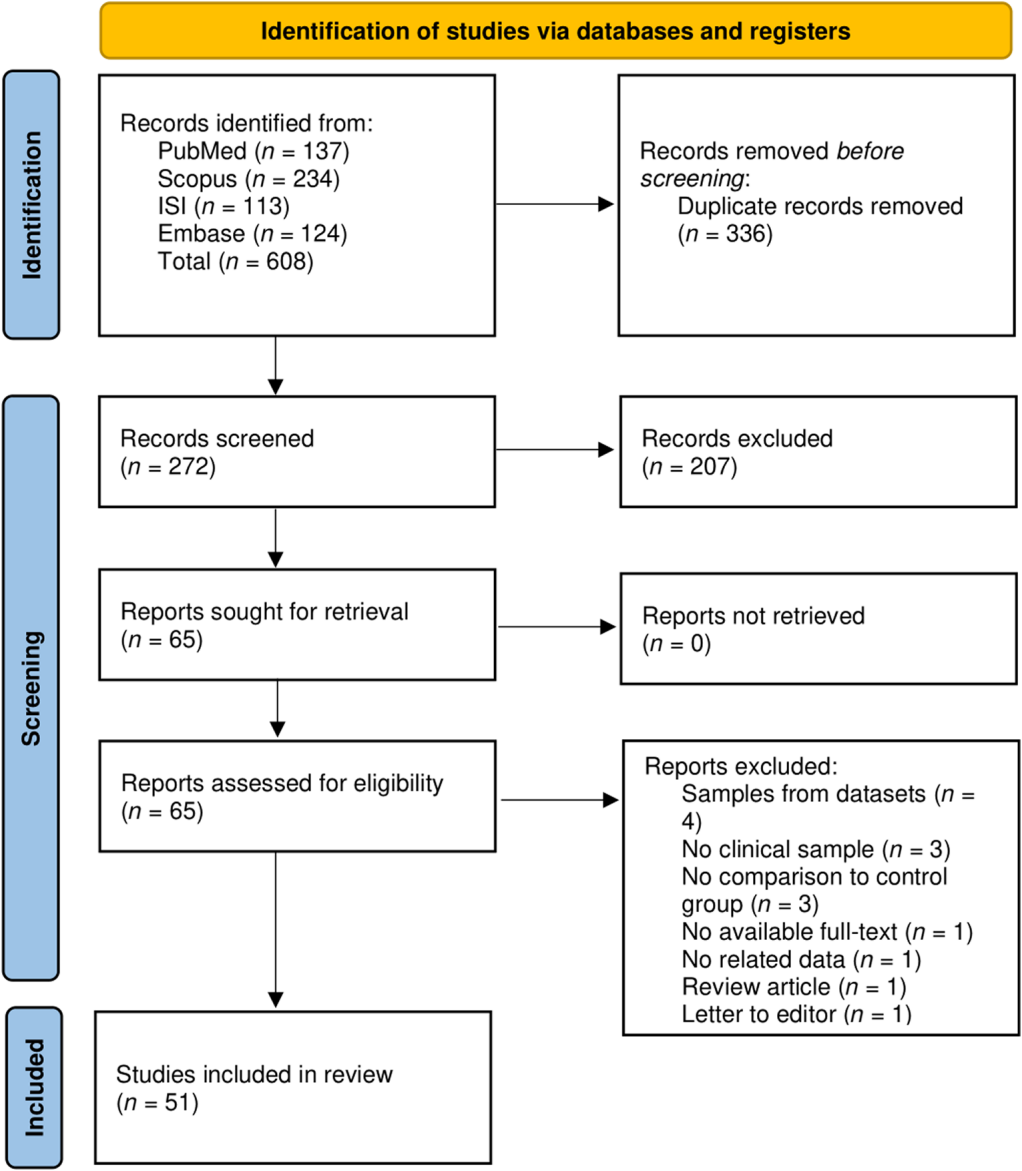
Flow diagram summarising the selection of eligible studies based on the PRISMA guidelines.

Our search identified 608 publications, including 124 articles from Embase, 113 from Web of Science, 137 from PubMed and 234 from Scopus. After removing duplicates, 272 records were screened through title and abstract, and 207 articles were removed. We reviewed the full‐text of 65 articles and excluded 14 articles (Supporting Information) due to the following reasons: (1) samples from datasets (*n* = 4); (2) no clinical sample (*n* = 3); (3) no comparison to the control group (*n* = 3); (4) no available full‐text (*n* = 1); (5) no related data (*n* = 1); (6) review article (*n* = 1); (7) letter to the editor (*n* = 1). Finally, 51 articles were included in our study. Figure 1 shows a flow diagram of study selection.

A total number of 2760 aggressive B‐cell NHL cases (2373 DLBCL, 320 MCL, 60 BL and 7 B‐lymphoblastic lymphomas [B‐LBL]) and 2552 controls (either adjacent normal tissue or samples from non‐NHL cases) were included in this study. Two studies did not report the exact number of cases and/or controls.^16^, ^17^ Forty‐four studies used lymph node tissue as their sample, and eight studies used serum/plasma/blood as their study sample. Whole transcriptome sequencing (WTS), copy number variation (CNV) and microarray expression was conducted by one, one and five studies, respectively. Overall, 79 lncRNA types were studied in aggressive B‐cell NHL patients. However, if we consider all results of WTS, CNV and microarray studies, more than 1000 lncRNAs had altered expression in aggressive B‐cell NHL patients.

All studies were ‘good’ or ‘fair’, given their NOS quality assessment scores. Details of quality assessment based on NOS scores are available in Table 1.

**TABLE 1:** Quality assessment of included studies.

ID	Author, year	Selection				Comparability	Exposure			Overall score
		Case definition	Representativeness	Selection of controls	Definition of controls		Ascertainment of exposure	Same method of ascertainment	Non‐response rate	
1	Chen, 2020	*			*	*	*	*	*	6
2	Conde, 2014	*	*	*	*	*	*	*	*	8
3	Deng, 2018	*		*	*	*	*	*	*	7
4	Doose, 2015	*				*	*	*	*	5
5	Esmeray Sönmez, 2022	*		*	*	*	*	*	*	7
6	Fan, 2019	*	*	*	*	*	*	*	*	8
7	Fu, 2021	*			*	*	*	*	*	6
8	Gao, 2017	*				*	*	*	*	5
9	Guo, 2020	*				*	*	*	*	5
10	Habieb, 2022	*	*	*	*	*	*	*	*	8
11	Hu, 2017	*			*	*	*	*	*	6
12	Hu, 2020	*			*	*	*	*	*	6
13	Huang, 2021	*			*	*	*	*	*	6
14	Li, 2021	*		*	*	*	*	*	*	7
15	Li, 2022	*			*	*	*	*	*	6
16	Liu, 2022	*	*	*	*	**	*	*	*	9
17	Meng, 2020	*				*	*	*	*	5
18	Peng, 2015	*			*	*	*	*	*	6
19	Peng, 2016	*	*	*	*	*	*	*	*	8
20	Peng, 2016	*	*	*	*	*	*	*	*	8
21	Peng, 2016	*	*	*	*	*	*	*	*	8
22	Qian, 2020	*			*	*	*	*	*	6
23	Qian, 2022	*	*		*	*	*	*	*	7
24	Senousy, 2021	*	*	*	*	**	*	*	*	9
25	Shi, 2019	*	*		*	*	*	*	*	7
26	Shu, 2022	*	*	*	*	*	*	*	*	8
27	Si, 2022	*			*	*	*	*	*	6
28	Song, 2020	*			*	*	*	*	*	6
29	Song, 2022	*			*	*	*	*	*	6
30	Tang X, 2020	*		*	*	*	*	*	*	7
31	Tao H, 2021	*			*	*	*	*	*	6
32	Tao S, 2022	*			*	*	*	*	*	6
33	Tian M, 2021	*			*	*	*	*	*	6
34	Tian Y 2021	*			*	*	*	*	*	6
35	Wang Q, 2019	*				*	*	*	*	5
36	Wang, 2016	*		*	*	*	*	*	*	7
37	Wang, 2017	*	*	*	*	*	*	*	*	8
38	Wen, 2019	*		*	*	*	*	*	*	7
39	Xing, 2022	*			*	*	*	*	*	6
40	Yan, 2016	*			*	*	*	*	*	6
41	Ye, 2022	*			*	*	*	*	*	6
42	Yuan, 2022	*				*	*	*	*	5
43	Zhang, 2019	*	*	*	*	**	*	*	*	9
44	Zhang, 2020	*			*	*	*	*	*	6
45	Zhao, 2019	*			*	*	*	*	*	6
46	Zhao, 2019	*			*	*	*	*	*	6
47	Zhao, 2020	*			*	*	*	*	*	6
48	Zhao, 2022	*			*	*	*	*	*	6
49	Zhou, 2022	*	*	*	*	*	*	*	*	8
50	Zhu, 2017	*			*	*	*	*	*	6
51	Zhu, 2019	*		*	*	*	*	*	*	7

### 3.2 Diffuse large B‐cell lymphoma

DLBCL was the most studied aggressive B‐cell NHL in this systematic review. Forty‐one studies investigated different lncRNAs in patients with DLBCL (Table 2). One study conducted a CNV analysis,^18^ and five studies^17^, ^19^, ^20^, ^21^, ^22^ investigated the microarray expression profile of lncRNAs in patients with DLBCL. In CNV analysis, partial duplication of lncRNA LOC283177 was detected in the blood of patients with DLBCL.^18^ Based on the microarray expression profiling of lncRNAs, NAALADL2‐AS2, HOTAIRM1, NR_026892, OR3A4, FIRRE, SNHG14, DUXAP8, LINC00473, SOX21‐AS1 and MIR503HG were upregulated but SMAD5‐AS1, ARHGEF35‐AS1 and OR2A1‐AS1 were downregulated.^17^, ^19^, ^20^, ^21^, ^22^ Furthermore, the upregulation of lncRNAs NAALADL2,^23^ FIRRE^24^ and SNHG14^25^ was also found in other studies.

**TABLE 2:** LncRNAs in DLBCL.

ID	Author, year	Method	Sample no.	lncRNA	Level	Marker type	Mechanism	Findings
1	Chen, 2020^43^	qRT‐PCR, tissue	80 DLBCL, 80 controls	SNHG12	Up	Prognostic/therapeutic target	SNHG12 overexpression promotes the growth, migration, and invasion of DLBCL cells/miR‐195 is a target of SNHG12/tumorigenesis	High SNHG12 expression was associated with poor OS and DFS
2	Conde, 2014^19^	CNV analysis, blood	242 DLBCL, 730 controls	LOC283177	Partial duplication	Diagnostic	NM	In CNV analysis, LOC283177 was the most significantly associated gene with DLBCL
3	Deng, 2018^46^	qRT‐PCR, tissue	64 DLBCL, 15 controls	NEAT1_1	Up	Prognostic	Cell proliferation, migration, apoptosis inhibition	The NEAT1_1 level was associated with cancer stage, IPI, extranodal site involvement, poor prognosis and drug response
4	Fu, 2021^40^	qRT‐PCR, tissue	50 DLBCL, adjacent normal tissue as controls	SBF2‐AS1	Up	Diagnostic	SBF2‐AS1 accelerated tumour growth via miR‐494‐3p/FGFR2 axis/migration/viability	–
5	Gao, 2017^20^	Microarray, qRT‐PCR, tissue	10 GCB‐DLBCL, 10 controls	NAALADL2‐AS2, HOTAIRM1, NR_026892	Up	Diagnostic	NM	From thousands of lncRNAs found in microarray, eight lncRNAs were selected for qRT‐PCR confirmation, and the expression of 5 lncRNAs was changed in clinical samples
				ARHGEF35‐AS1, OR2A1‐AS1	Down			
6	Habieb, 2022^27^	RT‐PCR, serum	60 DLBCL, 60 controls	HOTAIR, HOTTIP	Up	Diagnostic/prognostic/therapy response	NM	HOTTIP had a higher diagnostic value than HOTAIR alone or HOTAIR+HOTTIP, but HOTAIR had a higher value in identifying patients with an IPI >2. High HOTAIR and HOTTIP expressions were associated with advanced tumour stage, lower OS, worse PFS, and unresponsiveness to treatment
7	Hu, 2020^55^	qRT‐PCR, tissue	48 DLBCL, 14 controls	HCP5	Up	Therapeutic target	miR‐27b‐3p as a potential target of HCP5	Geniposide treatment reduced the expression of HCP5
8	Huang, 2021^47^	qRT‐PCR, tissue	87 DLBCL, adjacent normal tissue as controls	LINC00857	Up	Prognostic	LINC00857 contributed to DLBCL proliferation and lymphomagenesis via miR‐370‐3p/CBX3 axis regulation	LINC00857 overexpression was associated with a lower survival rate, more advanced tumour node metastasis and larger tumour size
9	Li, 2021^56^	qRT‐PCR, tissue	102 DLBCL, 102 controls	TUC338	Up	Therapy response/therapeutic target/therapy response	TUC338 bound to miR‐28‐5p and increased EGFR level, resulting in carcinogenic PI3K/AKT signalling activation, thereby facilitating DLBCL growth	High TUC338 expression was associated with advanced Ann Arbor stage, resistance to CHOP‐like treatment, worse OS and high IPI
10	Li, 2022^41^	RT‐PCR, blood	7 B‐LBL, 2 DLBCL, 3 BL, 12 controls	NORAD	Up	Diagnostic	NORAD regulated DLBCL cell growth and apoptosis via miR345‐3p/TRAF6/PI3K/Akt axis	NORAD levels were higher in the blood samples of B‐NHL patients than in the control group
11	Liu, 2022^25^	qRT‐PCR, tissue	53 DLBCL, 30 controls	FIRRE	Up	Prognostic/therapeutic target	Viability, transformation, and apoptosis regulation/through the interaction with PTBP1, FIRRE promoted the mRNA decay of Smurf2	FIRRE overexpression is associated with a higher ECOG performance score and a higher IPI
12	Meng, 2020^18^	Microarray, qRT‐PCR, tissue	DLBCL (NM)	OR3A4	Up	Prognostic	FOXM1‐induced upregulation of OR3A4 led to DLBCL occurrence via the Wnt/β‐catenin signalling pathway	204 lncRNAs were upregulated, and 113 lncRNAs were downregulated in DLBCL tissues. High OR3A4 expression was associated with poor prognosis
13	Peng, 2016^30^	qRT‐PCR, tissue	107 DLBCL, 46 controls	PEG10	Up	Diagnostic/prognostic/therapeutic target/therapy response	PEG10 knockdown led to growth arrest and cell apoptosis	High PEG10 was associated with B symptoms, IPI score, poor OS, CHOP‐like treatment and rituximab
14	Peng, 2016^32^	qRT‐PCR, tissue	87 DLBCL, 21 controls	LUNAR1	Up	Diagnostic/prognostic/therapy response	LUNAR1 knockdown suppressed cell proliferation of DLBCL by regulating E2F1, cyclin D1 and p21	Higher expression of LUNAR1 was significantly associated with stage, poor OS, rituximab and IPI
15	Peng, 2016^33^	qRT‐PCR, tissue	142 DLBCL, 60 controls	HULC	Up	Diagnostic/prognostic/therapeutic target/therapy response	HULC knockdown could arrest cell proliferation and induce apoptosis by suppressing cyclin D1 and Bcl‐2 in DLBCL cells	HULC overexpression was associated with Ann Arbor stages, B symptoms, poor OS, CHOP‐like treatment, rituximab and IPI
16	Peng, 2015^54^	qRT‐PCR, tissue	105 DLBCL, 36 controls	LincRNA‐p21	Down	Prognostic	Ectopic expression of lincRNA‐p21 inhibited cell proliferation and cycle progression and regulated cyclin D1, CDK4 and p21 expression	LincRNA‐p21 expression was associated with Ann Arbor stages, B symptoms, performance status, poor OS, IPI score and serum LDH
17	Qian, 2020^45^	qRT‐PCR, tissue	30 DLBCL, 30 controls	NEAT1	Up	Therapeutic target	NEAT1 acted as a ceRNA, regulating the miR‐34b‐5p‐GLI1 axis, further affecting the proliferation of DLBCL	–
18	Qian, 2022^49^	qRT‐PCR, tissue	90 PGI‐DLBCL, adjacent normal tissue as controls	MALAT1	Up	Prognostic/therapeutic target	NM	MALAT1 expression was increased in the non‐germinal center B‐cell‐like, advanced stage, and IPI score groups. High MALAT1 expression was associated with poor OS and PFS in PGI‐DLBCL patients
19	Senousy, 2021^28^	qRT‐PCR, plasma	84 DLBCL, 33 controls	HOTAIR, XIST	Up	Diagnostic/prognostic/therapy response	NM	Pretreatment plasma HOTAIR was higher, whereas GAS5 was lower in non‐responders than responders to R‐CHOP. Plasma GAS5 had a negative correlation with IPI, whereas HOTAIR had a positive correlation with performance status, denoting their prognostic potential
				GAS5	Down			
20	Shi, 2019^21^	Microarray, qRT‐PCR, tissue	70 DLBCL, 70 controls	FIRRE	Up	Prognostic	FIRRE activated the Wnt/β‐catenin signalling pathway to facilitate DLBCL cell growth via regulation of the nuclear translocation of β‐catenin	537 lncRNAs were differently expressed lncRNAs, among which 375 were upregulated lncRNAs in DLBCL patient samples. A high FIRRE level was associated with poor OS
21	Shu, 2022^50^	qRT‐PCR, tissue	100 DLBCL, 51 controls	HAGLROS	Up	Prognostic/therapeutic target	HAGLROS suppressed the expression of miR‐100, proliferation, migration and invasion	HAGLROS overexpression is associated with poor survival outcomes in DLBCL patients
22	Si, 2022^42^	qRT‐PCR, tissue	31 DLBCL, adjacent normal tissue as controls	SNHG12	Up	Therapeutic target	SNHG12 knockdown repressed cell proliferation and cell cycle, although heightened cell apoptosis in DLBCL cells. SNHG12 sponged miR‐494‐3p to adjust the CBX3	–
23	Song, 2022^36^	qRT‐PCR, tissue	8 DLBCL, 15 controls	TRERNA1	Up	Diagnostic/prognostic/therapeutic target	Decreased m6‐A methylation of TRERNA1 regulated by ALKBH5 regulates cell proliferation. TRERNA1 recruited EZH2 to epigenetically silence cyclin‐dependent kinases inhibitor p21 expression by H3K27me3 modification of its promoter region	TRERNA1 was associated with the poor prognosis of DLBCL patients
24	Song, 2020^57^	qRT‐PCR, tissue	26 DLBCL, 26 controls	DBH‐AS1	Up	Therapeutic target	proliferation, migration and invasion, DBH‐AS1/BUD13/FN1 axis	–
25	Tao, 2022^51^	qRT‐PCR, tissue	33 DLBCL, adjacent normal tissue as controls	PVT1	Up	Prognostic/therapeutic target	PVT1 sponged miR‐34b‐5p. Knockdown suppressed DLBCL cell proliferation but promoted apoptosis. PVT1/miR‐34b‐5p/Foxp1	–
26	Tian, 2021^52^	qRT‐PCR, tissue	48 DLBCL, adjacent normal tissue as controls	PCAT1	Up	Prognostic/therapeutic target	PCAT1 was upregulated in DLBCL and promoted cell proliferation, migration and invasion by regulating miR‐5083p/NFIB in DLBCL	PCAT1 expression was related to the clinical stage and IPI score
27	Tian, 2021^26^	qRT‐PCR, tissue	21 DLBCL, 21 controls	SNHG14	Up	Diagnostic/prognostic/therapeutic target	SNHG14 inhibits CTL activity and regulates DLBCL proliferation and apoptosis via miR‐152‐3p. SNHG14 promotes DLBCL progression via its sequestration of miR‐152‐3p, preventing its inhibition of the PD‐1/PD‐L1 checkpoint	–
28	Wang, 2019^48^	qRT‐PCR, tissue	37 DLBCL, adjacent normal tissue as controls	MALAT1	Up	Therapeutic target	MALAT1 sponged miR‐195 to regulate proliferation, apoptosis, migration and immune escape abilities of DLBCL by regulation of PD‐L1. MALAT 1 knockdown suppressed EMT‐like process via Ras/ERK signalling pathway	–
29	Wang, 2017^34^	qRT‐PCR, tissue, serum	68 DLBCL, adjacent normal tissue as controls	PANDA	Down	Diagnostic/prognostic/therapeutic target	PANDA was induced by p53, and p53 interacts with the promoter region of PANDA. PANDA inactivated the MAPK/ERK signalling pathway. PANDA suppresses proliferation and induces cell‐cycle arrest in DLBCL cells	Decreased serum PANDA level was correlated with poor clinical outcomes and OS in DLBCL patients
				TUG1	Up			
30	Xing, 2022^58^	qRT‐PCR, tissue	90 DLBCL, adjacent normal tissue as controls	SNHG5	Up	Therapeutic target	LncRNA SNHG5 acted as a ceRNA by binding with miR‐181‐5p in DLBC cells. LncRNA SNHG5 may promote the proliferation and migration of DLBCL cells via targeting miR‐181‐5p/XIAP. Knockdown of SNHG5 inhibited the proliferation, migration, and invasion	–
31	Yan, 2016^29^	qRT‐PCR, tissue	50 DLBCL, 20 controls	HOTAIR	Up	Prognostic/therapeutic target	PI3K/AKT/NF‐κB signalling pathway contributes to cell proliferation mediated by HOTAIR. Knockdown of HOTAIR led to growth inhibition, cell cycle arrest and apoptosis	HOTAIR was significantly correlated with tumour size, clinical stage, B symptoms and IPI scores. Higher expression levels of HOTAIR were correlated with poor prognosis
32	Ye, 2022^39^	qRT‐PCR, tissue	98 DLBCL, adjacent normal tissue as controls	ASHGA5P019110 (OR2A1‐ AS1)	Down	Prognostic/therapeutic target	NM	Reduced OR2A1‐ AS1 expression was linked to a shorter OS and PFS in DLBCL patients, especially those with GCB. Stratification analysis revealed the prognostic value of OR2A1‐AS in GCB‐ DLBCL but not in non‐GCB‐like‐DLBCL
33	Yuan, 2022^44^	qRT‐PCR, tissue	25 DLBCL, 25 controls	NEAT1	Up	Therapeutic target	NEAT1/miR‐495‐3p/PD‐L1 axis regulated the development of DLBCL. NEAT1 overexpression enhanced the cell viability and decreased apoptosis of DLBCL cells	–
34	Zhang, 2020^53^	qRT‐PCR, tissue	38 DLBCL, adjacent normal tissue as controls	UCA1	Up	Prognostic/therapeutic target	UCA1 regulates DLBCL cell progression by competitively binding with miR‐331‐3p. Knockdown of UCA1 inhibits cell proliferation, migration and invasion in DLBCL	–
35	Zhao, 2019^22^	Microarray, qRT‐PCR, tissue	3 DLBCL, 3 controls	SNHG14, DUXAP8, LINC00473, SOX21‐AS1, MIR503HG	Up	Therapeutic target	SNHG14 interacted with miR‐5590‐3p in DLBCL cells (reciprocal inhibition), promoting proliferation, invasion and EMT. Depletion of SNHG14 impaired the viability, colony generation and invasion of the DLBCL cell line	Targeting SNHG14 potentially improved the efficacy of immunotherapy in DLBCL through PD‐1/PD‐L1
36	Zhao, 2019^23^	Microarray, qRT‐PCR, tissue	11 DLBCL, 11 controls	SMAD5‐AS1	Down	Diagnostic/therapeutic target	SMAD5‐AS1/miR‐135b‐5p/APC axis regulated cell proliferation via the Wnt/β‐catenin signalling pathway. Up‐regulation of SMAD5‐AS1 inhibited cell viability and cycle and promoted apoptosis. Down‐regulation had the opposite effect	–
37	Zhao, 2022^35^	qRT‐PCR, tissue	50 DLBCL, 50 controls	TRIM52‐AS1	Up	Diagnostic/prognostic	miR‐577/IGFBP3/TRIM52 pathway, knockdown inhibited the proliferation of DLBCL cells and induced cell apoptosis	The high expression of TRIM52‐AS1 predicted poor Ann Arbor stage and presented B symptoms and a high IPI
38	Zhao, 2020^31^	qRT‐PCR, tissue	25 DLBCL, 25 controls	PEG10	Up	Diagnostic/therapeutic target	Sponging miR‐101‐3/cell proliferation and apoptosis/miR101‐3p/KIF2A axis/PEG10 deletion inhibited cell growth and metastasis and enhanced cell apoptosis in DLBCL	–
39	Zhou, 2022^37^	qRT‐PCR, tissue	30 DLBCL, 30 controls	CACNA1G‐AS1	Up	Diagnostic/prognostic/therapeutic target	Knockdown increased cytotoxicity and expedited apoptosis in DLBCL cells. microRNA (miR)‐3160‐5p is the downstream molecule for CACNA1G‐AS1	Expression of CACNA1G‐AS1 was associated with the clinical stage of DLBCL
40	Zhu, 2017^24^	qRT‐PCR, tissue	20 DLBCL, 9 controls	NAALADL2‐AS2, NONHSAT078790, NONHSAT102729	Up	Diagnostic/therapeutic target	NAALADL2‐AS2 has regulatory functions in p53, NF‐κB, JAK–STAT signalling pathways and in haematopoietic cell lineage	–
				NONHSAT120161, XIST	Down			
41	Zhu, 2019^38^	qRT‐PCR, tissue	48 DLBCL, 14 controls	SNHG16	Up	Diagnostic/prognostic	Cell cycle arrest at G0/G1 phase and tumour growth, SNHG16 knockdown inhibited cell proliferation and cell cycle progression and induced apoptosis of DLBCL cell. miR‐497‐5p/PIM1 axis	Advanced tumour stages showed higher levels of SNHG16. LncRNA SNHG16 is highly expressed in DLBCL

*Note*: See Supporting Information for the full form of lncRNA names.

Abbreviations: ALKBH5, AlkB homologue 5; APC, antigen‐presenting cell; Bcl‐2, B‐cell lymphoma 2; B‐LBL, B‐lymphoblastic lymphoma; BL, Burkitt's lymphoma; CBX3, chromobox protein homologue 3; CDK4, cyclin‐dependent kinase 4; ceRNA, competing endogenous RNA; CNV, copy number variation; CTL, cytotoxic T‐lymphocyte; DLBCL, diffuse large B‐cell lymphoma; DFS, disease‐free progression; E2F1, E2F transcription factor 1; ECOG, eastern cooperative oncology group; EGFR, epidermal growth factor receptor; EMT, epithelial–mesenchymal transition; ERK, extracellular signal‐regulated kinase; EZH2, enhancer of zeste homologue 2; FGFR2, fibroblast growth factor receptor 2; FN1, fibronectin 1; FOXM1, forkhead box protein M1; H3K27me3, trimethylation of lysine 27 on histone H3; GBC‐DLBCL, germinal‐center B‐cell‐like‐diffuse large B‐cell lymphoma; GLI1, glioma‐associated oncogene homologue 1; IGFBP3, insulin‐like growth factor binding protein 3; IPI, international prognostic index; JAK–STAT, janus kinase‐signal transducer and activator of transcription; KIF2A, kinesin superfamily protein 2A; LDH, lactate dehydrogenase; lincRNA, long intervening/intergenic noncoding RNA; MAPK, mitogen‐activated protein kinase; miR, microRNA; mRNA, messenger RNA; NF‐κB, nuclear factor kappa B; NFIB, nuclear factor 1 B‐type; NHL, non‐Hodgkin lymphoma; NM, not mentioned; OS, overall survival; PD‐1, programmed cell death protein 1; PD‐L1, programmed death‐ligand 1; PFS, progression‐free survival; PGI‐DLBCL; primary gastrointestinal diffuse large B‐cell lymphoma; PI3K/Akt, phosphatidylinositol‐3‐kinase and protein kinase B; PIM1, proviral integration site for Moloney murine leukaemia virus‐1; PTBP1, polypyrimidine tract‐binding protein 1; qRT‐PCR, real‐time reverse transcription polymerase chain reaction; RAS, reticular activating system; R‐CHOP, rituximab/cyclophosphamide/doxorubicin/prednisone/vincristine; SMURF2, SMAD ubiquitination regulatory factor 2; TRAF6, tumour necrosis factor receptor associated factor 6; XIAP, X‐linked inhibitor of apoptosis protein.

Among lncRNAs with diagnostic and prognostic values, HOTAIR,^26^, ^27^, ^28^ HOTTIP,^26^ PEG10,^29^, ^30^ LUNAR1,^31^ HULC,^32^ TUG1,^33^ TRIM52‐AS1,^34^ TRERNA1,^35^ CACNA1G‐AS1^36^ and SNHG16^37^ were upregulated, but NONHSAT120161,^23^ GAS5,^27^ OR2A1‐ AS1^19^, ^38^ and PANDA^33^ were downregulated. Two studies had controversial results regarding lncRNA XIST expression; Senousy et al.^27^ showed that lncRNA XIST is upregulated in the plasma of patients with DLBCL, but Zhu et al.^23^ found lower expression of lncRNA XIST in tissue samples from patients with DLBCL than normal controls. This controversy could be due to the difference in the sample type of the two studies.

Other lncRNAs with a diagnostic value and higher expression in patients with DLBCL include SBF2‐AS1,^39^ NORAD,^40^ NONHSAT078790^23^ and NONHSAT102729.^23^ LncRNAs with a prognostic value for DLBCL include SNHG12,^41^, ^42^ NEAT1,^43^, ^44^, ^45^ LINC00857,^46^ MALAT1,^47^, ^48^ HAGLROS,^49^ PVT1,^50^ PCAT1^51^ and UCA1,^52^ which were upregulated, and LincRNA‐p21,^53^ which was downregulated. Most of the lncRNAs mentioned above (diagnostic/prognostic/both) are suggested to be therapeutic targets for patients with DLBCL. In addition, lncRNAs HCP5,^54^ TUC338,^55^ DBH‐AS1^56^ and SNHG5^57^ are also among the potential targets for treating patients with DLBCL.

As mentioned above, lncRNA levels seem to be associated with overall survival (OS), disease‐free survival (DFS) and progression‐free survival (PFS). Table 3 summarizes the calculated hazard ratios (HRs) for the association of lncRNA level with OS, DFS and PFS in patients with DLBCL and MCL. HOTAIR had the highest HR (3.127, 95% confidence interval: 1.217–8.037) for OS in patients with DLBCL.

**TABLE 3:** Association of lncRNAs with survival.

Ref.	LncRNA	Overall survival (HR, 95% CI)	Disease‐free survival (HR, 95% CI)	Progression‐free survival (HR, 95% CI)
DLBCL				
^43^	SNHG12 (high vs. low)	1.232 (1.128–1.434)	1.332 (1.034–1.698)	
^30^	PEG10 (low vs. high)	0.5 (0.2–1.1)		
^32^	LUNAR1 (low vs. high)	0.955 (0.509–1.783)	0.686 (0.491–1.120)	
^33^	HULC (low vs. high)	0.738 (0.414–1.288)	0.482 (0.138–1.069)	
^54^	LincRNA‐p21 (low vs. high)	0.732 (0.518–1.074)		0.982 (0.733–1.373)
^49^	MALAT1 (low vs. high)	1.966 (1.024–3.771)		2.252 (1.187–4.274)
^50^	HAGLROS (high vs. low)	1.330 (1.218–1.624)		
^34^	PANDA (low vs. high)	1.893 (0.384–2.447)		
^29^	HOTAIR (high vs. low)	3.127 (1.217–8.037)		
^27^	HOTAIR	Mean: 13.16 months		Mean: 10.16 months
^27^	HOTTIP	Mean: 14.26 months		Mean: 10.91 months
^46^	NEAT1_1	Low vs. high: 23.7% vs. 46.2% death in 60 months		
MCL				
^63^	FOXP4‐AS1 (high vs. low)	1.496 (1.119–1.831)	1.496 (1.119–1.831)	

Abbreviations: CI, confidence interval; DLBCL, diffuse large B‐cell lymphoma; HR, hazard ratio; OS, overall survival.

Based on our results, lncRNAs could also be a biomarker for response to therapy in patients with DLBCL. High expression of lncRNAs TUC338,^55^ PEG10,^29^ HULC,^32^ HOTTIP^26^ and HOTAIR,^26^, ^27^ as well as low expression of lncRNA GAS5, were associated with poor response to CHOP‐like treatment ± rituximab. Moreover, using more than one lncRNA (e.g. HOTAIR+GAS5) as a marker of response to therapy could provide more accurate results.^27^

LncRNAs play their role in the pathogenesis of DLBCL through many mechanisms, including regulating the cell cycle, cell proliferation, apoptosis, transformation, viability and migration, methylation, tumour invasion, immune escape abilities and colony generation. Notably, many LncRNAs target microRNAs (miRNA) to affect the aforementioned cellular/molecular mechanisms (more details are available in Table 2).

### 3.3 Mantle cell lymphoma

Eight studies assessed the role of lncRNAs in patients with MCL^58^, ^59^, ^60^, ^61^, ^62^, ^63^, ^64^, ^65^ (Table 4). A WTS study demonstrated that 1067 lncRNAs were upregulated and 989 lncRNAs were downregulated in patients with MCL. Among the top 20 dysregulated lncRNAs, low expression of FTX was associated with poor OS. Based on this study lncRNAs play a role in MCL pathogenesis mostly via epidermal growth factor (EGF) receptor, Wnt and mammalian target of rapamycin (mTOR) signalling pathways.^58^ Among lncRNAs with a diagnostic value, lncRNAs GATA6‐AS^59^ and MORT^61^ were downregulated, and lncRNAs MANCR^64^ and LINK‐A^65^ were upregulated. These lncRNAs play their role in MCL pathogenesis by regulating survivin expression (LINK‐A), glucose uptake (GATA6‐AS), miRNA‐16 expression (MORT), inducing cell proliferation (MANCR, LINK‐A) and inhibiting apoptosis (LINK‐A).

**TABLE 4:** LncRNAs in MCL.

ID	Author, year	Method	Sample no.	lncRNA	Level	Marker type	Mechanism	Findings
1	Esmeray Sönmez, 2022^59^	WTS, tissue	32 MCL, 5 controls	Top 10: MIR100HG, LINC01268, FTX, ROR1‐AS1, DNM3OS, KCNQ1OT1, MAGI1‐IT1, NR2F2‐AS1, ADAMTS9‐AS2 and PCA3	Up	Prognostic	Mostly EGFR, Wnt and mTOR signalling pathways	WTS showed that 1067 lncRNAs were upregulated and 989 lncRNAs were downregulated in MCL cases. Low FTX expressions were associated with poor OS
				Top 10: LINC00877, SLC25A5‐AS1, ILF3‐DT, LRRC75A‐AS1, LINC00324, CD27‐AS1, ZFAS1, SNHG5, MIR762HG and SNRK‐AS1	Down			
2	Fan, 2019^60^	qRT‐PCR, plasma	47 MCL, 42 controls	GATA6‐AS	Down	Diagnostic	GATA6‐AS regulates glucose uptake, GLUT1 expression and GLUT1 involvement in the proliferation of MCL cells	Downregulation of GATA6‐AS has a potential diagnostic value in early‐stage MCL
3	Hu, 2017^61^	qRT‐PCR, tissue (mononuclear cells)	5 MCL, 5 controls	ROR1‐AS1, AC006196.1, RP11‐12A2.3, AF127936.5, AC010983.1, RP11‐436H11.6, GS1‐57 L11.1, RP11‐540A21.3, RP11‐436H11.3	Up	Therapy response	Overexpression of ROR1‐AS1 promoted tumour growth. ROR‐AS1 regulated gene transcription via associating with the PRC2 complex	ROR1‐AS1 overexpression decreased sensitivity to ibrutinib and dexamethasone
4	Tang, 2020^62^	qRT‐PCR, tissue	48 MCL, 42 controls	MORT	Down	Diagnostic/prognostic/therapeutic target	MORT could inhibit MCL cell proliferation and promote MCL cell apoptosis by upregulating miRNA‐16	Downregulation of MORT may assist in the early diagnosis of MCL
5	Tao, 2021^63^	qRT‐PCR, tissue	60 MCL, 53 controls	FOXP4‐AS1	Up	Prognostic/therapeutic target	FOXP4‐AS1 upregulates NACC1 by inhibiting miR‐423‐5p. Knockdown inhibits the proliferation, migration and invasion abilities of MCL cells	FOXP4‐AS1 expression predicts poor clinical outcomes
6	Wang, 2016^64^	qRT‐PCR, blood	40 MCL, 12 controls	MALAT1	Up	Prognostic/therapeutic target	MALAT1‐induced EZH2 recruitment is self‐enhanced through EZH2 phosphorylation at T350 in MCL. siRNA‐mediated knockdown of MALAT1, cell proliferation was decreased, and the percentage of apoptotic cells was significantly increased in MCL cells	The increased expression of MALAT1 was associated with the high‐risk group (by MIPI) and lower OS after current chemotherapy in patients with MCL
7	Wen, 2019^65^	qRT‐PCR, tissue	52 MCL, 38 controls	MANCR	Up	Diagnostic	Overexpression of MANCR mediated the overexpression of RUNX2. MANCR interacted with RUNX2 in the proliferation of MCL cells	–
8	Zhang, 2019^66^	qRT‐PCR, plasma	36 MCL, 32 controls	LINK‐A	Up	Diagnostic	LINK‐A upregulation causes cell proliferation, inhibits cell apoptosis, and upregulates survivin expression. LINK‐A lncRNA overexpression promoted cell proliferation	–

*Note*: See Supporting Information for the full form of lncRNA names.

Abbreviations: EGFR, epidermal growth factor receptor; EZH2, enhancer of zeste homologue 2; GLUT1, glucose transporter protein type 1; MCL, mantle cell lymphoma; mTOR, mammalian target of rapamycin; NACC1, nucleus accumbens associated 1; OS, overall survival; PRC2, polycomb repressive complex 2; qRT‐PCR, real‐time reverse transcription polymerase chain reaction; RUNX2, runt‐related transcription factor 2; siRNA, small interfering RNA; WTS, whole transcriptome sequencing.

Studies on lncRNAs MALAT1,^63^ FOXP4‐AS1^62^ and MORT^61^ have demonstrated that these lncRNAs have a prognostic value in MCL and could be a potential therapeutic target in patients with MCL. High expression of lncRNAs MALAT1 and FOXP4‐AS1 (HR: 1.496, 95% CI [1.119–1.831]) was associated with poor OS (Table 3). FOXP4‐AS1 causes MCL progression via inhibiting miR‐423‐5p, increasing the cell's migration, invasion, and proliferation abilities.^62^ LncRNA MALAT1 also affects cell proliferation and apoptosis via enhancer of zeste homologue 2 (EZH2) recruitment.^63^

A study by Hu et al. showed that lncRNA ROR1‐AS1 overexpression promotes tumour growth and decreases sensitivity to ibrutinib and dexamethasone in patients with MCL, highlighting its role as a marker for response to therapy.^60^

### 3.4 Burkitt's lymphoma

Three studies investigated the role of lncRNAs in patients with BL (Table 5).^16^, ^40^, ^66^ Guo et al. showed that lncRNA MCM3AP‐AS1 is upregulated in patients with BL and has a prognostic value; they found that MCM3AP‐AS1/miR‐15a/EIF4E axis regulates the chemoresistance of lymphoma cells and elevated levels of lncRNA MCM3AP‐AS1 is associated with poor OS, large tumour size and higher stages of the disease.^16^ Li et al. demonstrated that lncRNA NORAD is upregulated in the blood of patients with BL and has a diagnostic value.^40^ A study on lncRNA MINCR showed that it is a modulator of the MYC transcriptional programme, and its knockdown is associated with an impairment in cell cycle progression.^66^ It seems that lncRNAs MCM3AP‐AS1 and MINCR could be potential therapeutic targets for patients with BL.

**TABLE 5:** LncRNA in BL.

ID	Author, year	Method	Sample no.	lncRNA	Level	Marker type	Mechanism	Findings
1	Doose, 2015^67^	qRT‐PCR, tissue	16 BL, 4 controls	MINCR	Up	Therapeutic target	MINCR plays its role as a modulator of the MYC transcriptional programme. MINCR knockdown is associated with an impairment in cell cycle progression	–
2	Guo, 2020^17^	qRT‐PCR, tissue	41 BL, controls (NM)	MCM3AP‐AS1	Up	Prognostic/therapeutic target	MCM3AP‐AS1/miR‐15a/EIF4E axis regulated the chemoresistance of lymphoma cells	MCM3AP‐AS1 expression level is associated with tumour size and stage. MCM3AP‐AS1 favours doxorubicin‐induced chemoresistance via apoptosis inhibition and proliferation promotion. High expression of MCM3AP‐AS1 was associated with poor OS
3	Li, 2022^41^	RT‐PCR, blood	7 B‐LBL, 2 DLBCL, 3 BL, 12 controls	NORAD	Up	Diagnostic	NORAD regulated DLBCL cell growth and apoptosis via miR345‐3p/TRAF6/PI3K/Akt axis	NORAD levels were higher in the blood samples of B‐NHL patients than in the control group

*Note*: See Supporting Information for the full form of lncRNA names.

Abbreviations: B‐LBL, B‐lymphoblastic lymphoma; BL, Burkitt's lymphoma; DLBCL, diffuse large B‐cell lymphoma; NHL, non‐Hodgkin lymphoma; NM, not mentioned; OS, overall survival; PI3K/Akt, phosphatidylinositol‐3‐kinase and protein kinase B; qRT‐PCR, real‐time reverse transcription polymerase chain reaction; TRAF6, tumour necrosis factor receptor associated factor 6.

## 4 DISCUSSION

The present systematic review evaluated the alternations in the lncRNA expression profile in patients with aggressive B‐cell NHL to investigate their future potential in diagnosis, real‐time measurement of response to therapy and prognosis. In this study, we included the results of 51 original articles (2014–2023) on human subjects that evaluated the difference in expression levels of lncRNAs in samples from patients with DLBCL, MCL or BL compared to controls. Our result showed that lncRNAs have diagnostic and prognostic values and could be a potential therapeutic target in patients with aggressive B‐cell NHL.

The majority of RNAs, called non‐coding RNAs, do not code proteins; instead, they act as gene regulators.^67^, ^68^ MiRNAs have been the most studied non‐coding RNAs over the years. It has been revealed that miRNAs are involved in haematologic malignancies via various gene expression regulations.^69^ Also, miRNAs were introduced as excellent future biomarkers due to their high stability and tissue‐specific expression.^70^ LncRNAs are another class of non‐coding RNAs with more than 200 nucleotides. They are involved in distinct cellular functions and can be classified based on their wide range of functions. They can act as scaffolds (HOTAIR and NORAD), guides (MEG3), ribo‐activators (SRA), decoy (PANDA), competing endogenous RNAs (HULC) and precursors for small regulatory RNAs (MALAT1).^71^ Accumulative evidence has shown lncRNAs may also play a crucial role in the pathogenesis of different cancers, including B‐cell malignancies.^72^, ^73^ In recent years, more and more studies have been conducted evaluating the role of lncRNA as a novel biomarker in the development and progression of haematologic malignancies.

Currently, the gold standard for diagnosis of NHL is tissue biopsies of involved organs^74^ that are invasive and unsuitable for further follow‐ups. Circulating cancer‐associated molecules such as lncRNAs can be used as potential targets of liquid biopsy. Liquid biopsy, as a novel minimally invasive method for identifying various biomarkers, can provide an opportunistic window in diagnosing and monitoring NHL patients.^75^

Petri et al. analysed the expression of various lncRNAs in different stages of B‐cells, and their results suggest a key role for these molecules in the development of normal B‐cells.^9^ As it is believed that the factors leading to normal B‐cell formation are of great importance in B‐cell malignancies,^76^ studying the detailed functions of lncRNAs in B‐cell malignancies may guide us towards a better understanding of lymphomagenesis and developing novel lncRNA‐based therapies.

As mentioned before, we included studies on tissue samples from human subjects. However, several studies have identified differently expressed lncRNAs in aggressive B‐cell NHL using cell lines or data from datasets confirming the findings in tissue samples. For example, silencing lncRNA NEAT1 in BL cell lines of BJAB resulted in decreased viability, increased apoptosis and abnormal cell morphology, suggesting the possible role of lncRNA NEAT1 dysregulation in oncogenesis.^77^ LncRNA GAS5 is another lncRNA that has shown downregulation in DLBCL cell lines. Overexpression of GAS5 is associated with suppressing DLBCL progression and promoting programmed cell death.^78^ Also, some studies on datasets and cell lines have recognized lncRNAs in NHL cells without being evaluated in tissue samples. For instance, AFAP1‐AS1 had a high expression in DLBCL cell lines related to poor prognosis. This finding was confirmed by analysing The Cancer Genome Atlas (TCGA) database. Experiments have shown that AFAP1‐AS1 modulates gene expression by binding to different proteins promoting DLBCL progression and inhibiting apoptosis.^79^

Additionally, bioinformatics analyses have been used to implicit the possible involved pathways. Several studies showed that lncRNAs were involved in tumorigenesis pathways highlighting the potential value of lncRNAs as a therapeutic target.^80^, ^81^, ^82^ In the study by Li et al. on DLBCL datasets, lncRNA LINC01857 was upregulated, promoting cell proliferation and suppressing apoptosis in DLBCL cells. They revealed that LINC01857 acts as a sponge in the miR‐14‐3p/MAP4K4 axis that plays an important role in growth and apoptosis using bioinformatics study.^83^ A study by Xu et al., based on GEPIA analysis (47 DLBCL tissues and 337 blood tissues), showed upregulation of ARRDC1‐AS1 in DLBCL. Further analysis of cell lines demonstrated that ARRDC1‐AS1 leads to accelerated development of DLBCL via sponging miR‐2355‐5p. Their findings suggest targeting ARRDC1‐AS1 can be a potential therapeutic strategy for DLBCL.^84^

Several studies used more than one lncRNA as a biomarker to predict the prognosis of patients with DLBCL. Gao et al. analysed 623 DLBCL samples and 157 controls from multiple datasets. They constructed a 6‐lncRNA (SNHG26, RPARP‐AS1, AC244090.1, PRKCQ‐AS1, AC018521.5 and AC023590.1) scoring system predicting disease outcome; upregulation of two SNHG26 and RPARP‐AS1 was associated with poor prognosis, while high expression of the other four lncRNAs was related to a better prognosis.^85^ Moreover, Wang et al. have designed a 9‐lncRNA signature to predict the prognosis of DLBCL.^86^

Based on our results, another potential clinical use of lncRNAs could be predicting the response rate in distinct therapies in patients with aggressive B‐cell NHL. A study on BL xenografts showed that the deletion of DLEU1 was related to shorter survival and chemoimmunotherapy resistance.^87^

To the best of our knowledge, this is the first study that systematically reviews the role of lncRNAs in aggressive B‐cell NHL. However, there were some limitations in our study. Due to the limited number of articles evaluating a specific lncRNA and the heterogeneity of studies, we could not perform a meta‐analysis. Besides, the sample size was small in some of the included studies, which could affect the study results.

## 5 CONCLUSION

In conclusion, the evidence highlighting the emerging role of lncRNAs in the initiation and progression of aggressive B‐cell NHL suggests lncRNAs as novel biomarkers for diagnostic, prognostic, and therapeutic purposes. Further studies should be conducted to evaluate lncRNA‐based therapies.

## AUTHOR CONTRIBUTIONS

**Shaghayegh Khanmohammadi:** Conceptualization (lead); data curation (equal); formal analysis (equal); investigation (equal); methodology (equal); project administration (lead); supervision (lead); validation (equal); visualization (equal); writing – original draft (equal); writing – review and editing (equal). **Parisa Fallahtafti:** Conceptualization (supporting); data curation (equal); formal analysis (equal); investigation (equal); methodology (equal); project administration (supporting); supervision (equal); validation (equal); visualization (equal); writing – original draft (equal); writing – review and editing (equal).

## CONFLICT OF INTEREST STATEMENT

The authors confirm that there are no conflicts of interest.

## Supporting information


Supplementary material

## Data Availability

Data sharing not applicable—no new data generated, or the article describes entirely theoretical research.
