# Supplementary material for: Long non‐coding RNA as a novel biomarker and therapeutic target in aggressive B‐cell non‐Hodgkin lymphoma: A systematic review
Source: J Cell Mol Med. 2023 May 29;27(14):1928–46. doi: 10.1111/jcmm.17795 (PMC10339099; doi:10.1111/jcmm.17795)
Supplement: Supplementary file 1 — Supplementary material [file JCMM-27-1928-s001.pdf]

Search strategy:

PubMed:

(“Lymphoma, Large B-Cell, Diffuse”[MeSH] OR “Mantle cell lymphoma”[MeSH] OR “Burkitt lymphoma”[MeSH] OR “Lymphoma, Histiocytic”[tiab] OR “Histiocytic Lymphomas”[tiab] OR “Lymphoma, Large Lymphoid, Diffuse”[tiab] OR “Lymphoma, Histiocytic, Diffuse”[tiab] OR “Lymphoma, Large Cell, Diffuse”[tiab] OR “Lymphoma, Diffuse Large-Cell”[tiab] OR “Lymphoma, Diffuse Large Cell”[tiab] OR “Diffuse Large-Cell Lymphoma”[tiab] OR “Diffuse Large Cell Lymphoma”[tiab] OR “Diffuse Large-Cell Lymphomas”[tiab] OR “Diffuse, Large B-Cell, Lymphoma”[tiab] OR “Histiocytic Lymphoma”[tiab] OR “Histiocytic Lymphoma, Diffuse”[tiab] OR “Diffuse Histiocytic Lymphoma”[tiab] OR “Diffuse Histiocytic Lymphomas”[tiab] OR “Lymphoma, Diffuse Histiocytic”[tiab] OR “Large Lymphoid Lymphoma, Diffuse”[tiab] OR “Large-Cell Lymphoma, Diffuse”[tiab] OR “Large Cell Lymphoma, Diffuse”[tiab] OR “Diffuse Large B-Cell Lymphoma”[tiab] OR “Diffuse Large B Cell Lymphoma”[tiab] OR “Lymphoma, Large-Cell, Diffuse”[tiab] OR “Lymphoma, Mantle Cell”[tiab] OR “Lymphomas, Mantle-Cell”[tiab] OR “Mantle-Cell Lymphomas”[tiab] OR “Lymphocytic Lymphoma, Diffuse, Poorly Differentiated”[tiab] OR “Lymphocytic Lymphoma, Diffuse, Poorly-Differentiated”[tiab] OR “Mantle-Zone Lymphoma”[tiab] OR “Lymphoma, Mantle-Zone”[tiab] OR “Lymphomas, Mantle-Zone”[tiab] OR “Mantle Zone Lymphoma”[tiab] OR “Mantle-Zone Lymphomas”[tiab] OR “Lymphoma, Lymphocytic, Diffuse, Intermediate Differentiated”[tiab] OR “Lymphoma, Lymphocytic, Diffuse, Poorly-Differentiated”[tiab] OR “Lymphoma, Small-Cell, Centrocytic”[tiab] OR “Mantle-Cell Lymphoma”[tiab] OR “Mantle Cell Lymphoma”[tiab] OR “Diffuse Lymphocytic Lymphoma, Poorly-Differentiated”[tiab] OR “Diffuse Lymphocytic Lymphoma, Poorly Differentiated”[tiab] OR “Lymphoma, Centrocytic Small-Cell”[tiab] OR “Centrocytic Small-Cell Lymphoma”[tiab] OR “Centrocytic Small-Cell Lymphomas”[tiab] OR “Lymphoma, Centrocytic Small Cell”[tiab] OR “Lymphomas, Centrocytic Small-Cell”[tiab] OR “Small-Cell Lymphoma, Centrocytic”[tiab] OR “Small-Cell Lymphomas, Centrocytic”[tiab] OR “Lymphoma, Lymphocytic, Intermediate”[tiab] OR “Burkitt Tumor”[tiab] OR “Burkitt Lymphoma”[tiab] OR “Tumor, Burkitt”[tiab] OR “Burkitt's Tumor”[tiab] OR “Burkitts Tumor”[tiab] OR “Tumor, Burkitt's”[tiab] OR “Burkitt's Lymphoma”[tiab] OR “Burkitts Lymphoma”[tiab] OR “Lymphoma, Burkitt's”[tiab] OR “Lymphoma, Burkitt”[tiab] OR “Burkitt Leukemia”[tiab] OR “Leukemia, Burkitt”[tiab] OR “Burkitt Cell Leukemia”[tiab] OR “Leukemia, Burkitt Cell”[tiab] OR “Leukemia, Lymphocytic, L3”[tiab] OR “Lymphocytic Leukemia, L3”[tiab] OR “L3 Lymphocytic Leukemia”[tiab] OR “L3 Lymphocytic Leukemias”[tiab] OR “Leukemia, L3 Lymphocytic”[tiab] OR “Burkitt's Leukemia”[tiab] OR “Burkitts Leukemia”[tiab] OR “Leukemia, Burkitt's”[tiab] OR “Leukemia, Lymphoblastic, Burkitt-Type”[tiab] OR “African Lymphoma”[tiab] OR “Lymphoma, African”[tiab]) AND (“Noncoding RNA, Long”[tiab] OR “lncRNA”[tiab] OR “Long ncRNA”[tiab] OR “ncRNA, Long”[tiab] OR “RNA, Long Non-Translated”[tiab] OR “Long Non-Translated RNA”[tiab] OR “Non-Translated RNA, Long”[tiab] OR “RNA, Long Non Translated”[tiab] OR “Long Non-Coding RNA”[tiab] OR “Long Non Coding RNA”[tiab] OR “Non-Coding RNA, Long”[tiab] OR “RNA, Long Non-Coding”[tiab] OR “Long Non-Protein-Coding RNA”[tiab] OR “Long Non Protein Coding RNA”[tiab] OR “Non-Protein-Coding RNA, Long”[tiab] OR “RNA, Long Non-

Protein-Coding"[tiab] OR "Long Noncoding RNA"[tiab] OR "RNA, Long Untranslated"[tiab] OR "Long Untranslated RNA"[tiab] OR "Untranslated RNA, Long"[tiab] OR "Long ncRNAs"[tiab] OR "ncRNAs, Long"[tiab] OR "Long Intergenic Non-Protein Coding RNA"[tiab] OR "Long Intergenic Non Protein Coding RNA"[tiab] OR "LincRNAs"[tiab] OR "LINC RNA"[tiab] OR "LincRNA"[tiab] OR RNA, Long Noncoding[MeSH])

ISI:

TS=("Lymphoma, Histiocytic" OR "Histiocytic Lymphomas" OR "Lymphoma, Large Lymphoid, Diffuse" OR "Lymphoma, Histiocytic, Diffuse" OR "Lymphoma, Large Cell, Diffuse" OR "Lymphoma, Diffuse Large-Cell" OR "Lymphoma, Diffuse Large Cell" OR "Diffuse Large-Cell Lymphoma" OR "Diffuse Large Cell Lymphoma" OR "Diffuse Large-Cell Lymphomas" OR "Diffuse, Large B-Cell, Lymphoma" OR "Histiocytic Lymphoma" OR "Histiocytic Lymphoma, Diffuse" OR "Diffuse Histiocytic Lymphoma" OR "Diffuse Histiocytic Lymphomas" OR "Lymphoma, Diffuse Histiocytic" OR "Large Lymphoid Lymphoma, Diffuse" OR "Large-Cell Lymphoma, Diffuse" OR "Large Cell Lymphoma, Diffuse" OR "Diffuse Large B-Cell Lymphoma" OR "Diffuse Large B Cell Lymphoma" OR "Lymphoma, Large-Cell, Diffuse" OR "Lymphoma, Mantle Cell" OR "Lymphomas, Mantle-Cell" OR "Mantle-Cell Lymphomas" OR "Lymphocytic Lymphoma, Diffuse, Poorly Differentiated" OR "Lymphocytic Lymphoma, Diffuse, Poorly-Differentiated" OR "Mantle-Zone Lymphoma" OR "Lymphoma, Mantle-Zone" OR "Lymphomas, Mantle-Zone" OR "Mantle Zone Lymphoma" OR "Mantle-Zone Lymphomas" OR "Lymphoma, Lymphocytic, Diffuse, Intermediate Differentiated" OR "Lymphoma, Lymphocytic, Diffuse, Poorly-Differentiated" OR "Lymphoma, Small-Cell, Centrocytic" OR "Mantle-Cell Lymphoma" OR "Mantle Cell Lymphoma" OR "Diffuse Lymphocytic Lymphoma, Poorly-Differentiated" OR "Diffuse Lymphocytic Lymphoma, Poorly Differentiated" OR "Lymphoma, Centrocytic Small-Cell" OR "Centrocytic Small-Cell Lymphoma" OR "Centrocytic Small-Cell Lymphomas" OR "Lymphoma, Centrocytic Small Cell" OR "Lymphomas, Centrocytic Small-Cell" OR "Small-Cell Lymphoma, Centrocytic" OR "Small-Cell Lymphomas, Centrocytic" OR "Lymphoma, Lymphocytic, Intermediate" OR "Burkitt Tumor" OR "Burkitt Lymphoma" OR "Tumor, Burkitt" OR "Burkitt's Tumor" OR "Burkitts Tumor" OR "Tumor, Burkitt's" OR "Burkitt's Lymphoma" OR "Burkitts Lymphoma" OR "Lymphoma, Burkitt's" OR "Lymphoma, Burkitt" OR "Burkitt Leukemia" OR "Leukemia, Burkitt" OR "Burkitt Cell Leukemia" OR "Leukemia, Burkitt Cell" OR "Leukemia, Lymphocytic, L3" OR "Lymphocytic Leukemia, L3" OR "L3 Lymphocytic Leukemia" OR "L3 Lymphocytic Leukemias" OR "Leukemia, L3 Lymphocytic" OR "Burkitt's Leukemia" OR "Burkitts Leukemia" OR "Leukemia, Burkitt's" OR "Leukemia, Lymphoblastic, Burkitt-Type" OR "African Lymphoma" OR "Lymphoma, African") AND TS=("Noncoding RNA, Long" OR "lncRNA" OR "Long ncRNA" OR "ncRNA, Long" OR "RNA, Long Non-Translated" OR "Long Non-Translated RNA" OR "Non-Translated RNA, Long" OR "RNA, Long Non Translated" OR "Long Non-Coding RNA" OR "Long Non Coding RNA" OR "Non-Coding RNA, Long" OR "RNA, Long Non-Coding" OR "Long Non-Protein-Coding RNA" OR "Long Non Protein Coding RNA" OR "Non-Protein-Coding RNA, Long" OR "RNA, Long Non-Protein-Coding" OR "Long Noncoding RNA" OR

“RNA, Long Untranslated” OR “Long Untranslated RNA” OR “Untranslated RNA, Long” OR “Long ncRNAs” OR “ncRNAs, Long” OR “Long Intergenic Non-Protein Coding RNA” OR “Long Intergenic Non Protein Coding RNA” OR “LincRNAs” OR “LINC RNA” OR “LincRNA”)

SCOPUS:

TITLE-ABS-KEY(“Lymphoma, Histiocytic” OR “Histiocytic Lymphomas” OR “Lymphoma, Large Lymphoid, Diffuse” OR “Lymphoma, Histiocytic, Diffuse” OR “Lymphoma, Large Cell, Diffuse” OR “Lymphoma, Diffuse Large-Cell” OR “Lymphoma, Diffuse Large Cell” OR “Diffuse Large-Cell Lymphoma” OR “Diffuse Large Cell Lymphoma” OR “Diffuse Large-Cell Lymphomas” OR “Diffuse, Large B-Cell, Lymphoma” OR “Histiocytic Lymphoma” OR “Histiocytic Lymphoma, Diffuse” OR “Diffuse Histiocytic Lymphoma” OR “Diffuse Histiocytic Lymphomas” OR “Lymphoma, Diffuse Histiocytic” OR “Large Lymphoid Lymphoma, Diffuse” OR “Large-Cell Lymphoma, Diffuse” OR “Large Cell Lymphoma, Diffuse” OR “Diffuse Large B-Cell Lymphoma” OR “Diffuse Large B Cell Lymphoma” OR “Lymphoma, Large-Cell, Diffuse” OR “Lymphoma, Mantle Cell” OR “Lymphomas, Mantle-Cell” OR “Mantle-Cell Lymphomas” OR “Lymphocytic Lymphoma, Diffuse, Poorly Differentiated” OR “Lymphocytic Lymphoma, Diffuse, Poorly-Differentiated” OR “Mantle-Zone Lymphoma” OR “Lymphoma, Mantle-Zone” OR “Lymphomas, Mantle-Zone” OR “Mantle Zone Lymphoma” OR “Mantle-Zone Lymphomas” OR “Lymphoma, Lymphocytic, Diffuse, Intermediate Differentiated” OR “Lymphoma, Lymphocytic, Diffuse, Poorly-Differentiated” OR “Lymphoma, Small-Cell, Centrocytic” OR “Mantle-Cell Lymphoma” OR “Mantle Cell Lymphoma” OR “Diffuse Lymphocytic Lymphoma, Poorly-Differentiated” OR “Diffuse Lymphocytic Lymphoma, Poorly Differentiated” OR “Lymphoma, Centrocytic Small-Cell” OR “Centrocytic Small-Cell Lymphoma” OR “Centrocytic Small-Cell Lymphomas” OR “Lymphoma, Centrocytic Small Cell” OR “Lymphomas, Centrocytic Small-Cell” OR “Small-Cell Lymphoma, Centrocytic” OR “Small-Cell Lymphomas, Centrocytic” OR “Lymphoma, Lymphocytic, Intermediate” OR “Burkitt Tumor” OR “Burkitt Lymphoma” OR “Tumor, Burkitt” OR “Burkitt's Tumor” OR “Burkitts Tumor” OR “Tumor, Burkitt's” OR “Burkitt's Lymphoma” OR “Burkitts Lymphoma” OR “Lymphoma, Burkitt's” OR “Lymphoma, Burkitt” OR “Burkitt Leukemia” OR “Leukemia, Burkitt” OR “Burkitt Cell Leukemia” OR “Leukemia, Burkitt Cell” OR “Leukemia, Lymphocytic, L3” OR “Lymphocytic Leukemia, L3” OR “L3 Lymphocytic Leukemia” OR “L3 Lymphocytic Leukemias” OR “Leukemia, L3 Lymphocytic” OR “Burkitt's Leukemia” OR “Burkitts Leukemia” OR “Leukemia, Burkitt's” OR “Leukemia, Lymphoblastic, Burkitt-Type” OR “African Lymphoma” OR “Lymphoma, African”) AND TITLE-ABS-KEY(“Noncoding RNA, Long” OR “lncRNA” OR “Long ncRNA” OR “ncRNA, Long” OR “RNA, Long Non-Translated” OR “Long Non-Translated RNA” OR “Non-Translated RNA, Long” OR “RNA, Long Non Translated” OR “Long Non-Coding RNA” OR “Long Non Coding RNA” OR “Non-Coding RNA, Long” OR “RNA, Long Non-Coding” OR “Long Non-Protein-Coding RNA” OR “Long Non Protein Coding RNA” OR “Non-Protein-Coding RNA, Long” OR “RNA, Long Non-Protein-Coding” OR “Long Noncoding RNA” OR “RNA, Long Untranslated” OR “Long Untranslated RNA” OR “Untranslated RNA, Long” OR “Long ncRNAs” OR “ncRNAs, Long” OR “Long Intergenic Non-

Protein Coding RNA” OR “Long Intergenic Non Protein Coding RNA” OR “LincRNAs” OR “LINC RNA” OR “LincRNA”)

EMBASE:

(“Lymphoma, Histiocytic”:ti,ab,kw OR “Histiocytic Lymphomas”:ti,ab,kw OR “Lymphoma, Large Lymphoid, Diffuse”:ti,ab,kw OR “Lymphoma, Histiocytic, Diffuse”:ti,ab,kw OR “Lymphoma, Large Cell, Diffuse”:ti,ab,kw OR “Lymphoma, Diffuse Large-Cell”:ti,ab,kw OR “Lymphoma, Diffuse Large Cell”:ti,ab,kw OR “Diffuse Large-Cell Lymphoma”:ti,ab,kw OR “Diffuse Large Cell Lymphoma”:ti,ab,kw OR “Diffuse Large-Cell Lymphomas”:ti,ab,kw OR “Diffuse, Large B-Cell, Lymphoma”:ti,ab,kw OR “Histiocytic Lymphoma”:ti,ab,kw OR “Histiocytic Lymphoma, Diffuse”:ti,ab,kw OR “Diffuse Histiocytic Lymphoma”:ti,ab,kw OR “Diffuse Histiocytic Lymphomas”:ti,ab,kw OR “Lymphoma, Diffuse Histiocytic”:ti,ab,kw OR “Large Lymphoid Lymphoma, Diffuse”:ti,ab,kw OR “Large-Cell Lymphoma, Diffuse”:ti,ab,kw OR “Large Cell Lymphoma, Diffuse”:ti,ab,kw OR “Diffuse Large B-Cell Lymphoma”:ti,ab,kw OR “Diffuse Large B Cell Lymphoma”:ti,ab,kw OR “Lymphoma, Large-Cell, Diffuse”:ti,ab,kw OR “Lymphoma, Mantle Cell”:ti,ab,kw OR “Lymphomas, Mantle-Cell”:ti,ab,kw OR “Mantle-Cell Lymphomas”:ti,ab,kw OR “Lymphocytic Lymphoma, Diffuse, Poorly Differentiated”:ti,ab,kw OR “Lymphocytic Lymphoma, Diffuse, Poorly-Differentiated”:ti,ab,kw OR “Mantle-Zone Lymphoma”:ti,ab,kw OR “Lymphoma, Mantle-Zone”:ti,ab,kw OR “Lymphomas, Mantle-Zone”:ti,ab,kw OR “Mantle Zone Lymphoma”:ti,ab,kw OR “Mantle-Zone Lymphomas”:ti,ab,kw OR “Lymphoma, Lymphocytic, Diffuse, Intermediate Differentiated”:ti,ab,kw OR “Lymphoma, Lymphocytic, Diffuse, Poorly-Differentiated”:ti,ab,kw OR “Lymphoma, Small-Cell, Centrocytic”:ti,ab,kw OR “Mantle-Cell Lymphoma”:ti,ab,kw OR “Mantle Cell Lymphoma”:ti,ab,kw OR “Diffuse Lymphocytic Lymphoma, Poorly-Differentiated”:ti,ab,kw OR “Diffuse Lymphocytic Lymphoma, Poorly Differentiated”:ti,ab,kw OR “Lymphoma, Centrocytic Small-Cell”:ti,ab,kw OR “Centrocytic Small-Cell Lymphoma”:ti,ab,kw OR “Centrocytic Small-Cell Lymphomas”:ti,ab,kw OR “Lymphoma, Centrocytic Small Cell”:ti,ab,kw OR “Lymphomas, Centrocytic Small-Cell”:ti,ab,kw OR “Small-Cell Lymphoma, Centrocytic”:ti,ab,kw OR “Small-Cell Lymphomas, Centrocytic”:ti,ab,kw OR “Lymphoma, Lymphocytic, Intermediate”:ti,ab,kw OR “Burkitt Tumor”:ti,ab,kw OR “Burkitt Lymphoma”:ti,ab,kw OR “Tumor, Burkitt”:ti,ab,kw OR “Burkitt's Tumor”:ti,ab,kw OR “Burkitts Tumor”:ti,ab,kw OR “Tumor, Burkitt's”:ti,ab,kw OR “Burkitt's Lymphoma”:ti,ab,kw OR “Burkitts Lymphoma”:ti,ab,kw OR “Lymphoma, Burkitt's”:ti,ab,kw OR “Lymphoma, Burkitt”:ti,ab,kw OR “Burkitt Leukemia”:ti,ab,kw OR “Leukemia, Burkitt”:ti,ab,kw OR “Burkitt Cell Leukemia”:ti,ab,kw OR “Leukemia, Burkitt Cell”:ti,ab,kw OR “Leukemia, Lymphocytic, L3”:ti,ab,kw OR “Lymphocytic Leukemia, L3”:ti,ab,kw OR “L3 Lymphocytic Leukemia”:ti,ab,kw OR “L3 Lymphocytic Leukemias”:ti,ab,kw OR “Leukemia, L3 Lymphocytic”:ti,ab,kw OR “Burkitt's Leukemia”:ti,ab,kw OR “Burkitts Leukemia”:ti,ab,kw OR “Leukemia, Burkitt's”:ti,ab,kw OR “Leukemia, Lymphoblastic, Burkitt-Type”:ti,ab,kw OR “African Lymphoma”:ti,ab,kw OR “Lymphoma, African”:ti,ab,kw) AND (“Noncoding RNA, Long”:ti,ab,kw OR “lncRNA”:ti,ab,kw OR “Long ncRNA”:ti,ab,kw OR “ncRNA, Long”:ti,ab,kw

OR “RNA, Long Non-Translated”:ti,ab,kw OR “Long Non-Translated RNA”:ti,ab,kw OR “Non-Translated RNA, Long”:ti,ab,kw OR “RNA, Long Non Translated”:ti,ab,kw OR “Long Non-Coding RNA”:ti,ab,kw OR “Long Non Coding RNA”:ti,ab,kw OR “Non-Coding RNA, Long”:ti,ab,kw OR “RNA, Long Non-Coding”:ti,ab,kw OR “Long Non-Protein-Coding RNA”:ti,ab,kw OR “Long Non Protein Coding RNA”:ti,ab,kw OR “Non-Protein-Coding RNA, Long”:ti,ab,kw OR “RNA, Long Non-Protein-Coding”:ti,ab,kw OR “Long Noncoding RNA”:ti,ab,kw OR “RNA, Long Untranslated”:ti,ab,kw OR “Long Untranslated RNA”:ti,ab,kw OR “Untranslated RNA, Long”:ti,ab,kw OR “Long ncRNAs”:ti,ab,kw OR “ncRNAs, Long”:ti,ab,kw OR “Long Intergenic Non-Protein Coding RNA”:ti,ab,kw OR “Long Intergenic Non Protein Coding RNA”:ti,ab,kw OR “LincRNAs”:ti,ab,kw OR “LINC RNA”:ti,ab,kw OR “LincRNA”:ti,ab,kw)

Abbreviations (only lncRNAs):

SNHG12: small nucleolar RNA host gene 12; MIR100HG: Mir-100-Let-7a-2-Mir-125b-1 cluster host gene; FTX: five prime to XIST; DNM3OS: dynamin 3 opposite strand; MAGI1-IT1: membrane-associated guanylate kinase inverted 1; NR2F2-AS1: NR2F2 antisense RNA 1; PCA3: prostate cancer antigen 3; LINC00877: long intergenic non-protein coding RNA 877; ILF3-DT: ILF3 divergent transcript; LRRC75A-AS1: leucine-rich repeat-containing protein 75A antisense RNA 1; LINC00324: Long intergenic non–protein-coding RNA 324; CD27-AS1: CD27 antisense RNA 1; ZFAS1: zinc finger antisense 1; SNHG5: small nucleolar RNA host gene 5; MIR762HG: MIR762 host gene; SNRK-AS1: SNRK Antisense RNA 1; HOTTIP: HOXA transcript at the distal tip; GATA6-AS: GATA binding protein 6 antisense RNA 1; SBF2-AS1: SET binding factor 2-antisense strand 1; MCM3AP-AS1: mini-chromosome maintenance complex component 3 associated protein antisense 1; EIF4E: eukaryotic translation initiation factor 4E; ROR1-AS1: ROR1 antisense RNA 1; HCP5: histocompatibility leukocyte antigen complex P5; LINC00857: Long Intergenic Non-Protein Coding RNA 857; TUC338: transcribed ultra-conserved region 338; NORAD: non-coding RNA activated by DNA damage; FIRRE: firre intergenic repeating RNA element; OR3A4: olfactory receptor, family 3, subfamily A, member 4; PEG10: paternally expressed gene 10; LUNAR1: leukemia-associated non-coding IGF1R activator RNA 1; HULC: highly upregulated in liver cancer; NEAT1: nuclear paraspeckle assembly transcript 1; HAGLROS: HOXD antisense growth-associated long noncoding RNA; SNHG12: small nucleolar RNA host gene 12; TRERNA1: translation regulatory long non-coding RNA 1; DBH-AS1: DBH antisense RNA1; MORT: mortal obligate RNA transcript; FOXP4-AS1: forkhead box P4 antisense RNA 1; PVT1: plasmacytoma variant translocation 1; PCAT1: prostate cancer associated transcript-1; SNHG14: small nucleolar host gene 14; DUXAP8: derived lncRNA double homeobox A pseudogene 8; SOX21-AS1: SOX21 Antisense Divergent Transcript 1; LINC00473: long intergenic non-coding RNA 00473; MIR503HG: MIR503 host gene; OR2A1-AS1: OR2A1 Antisense RNA 1; MALAT1: metastasis-associated lung adenocarcinoma transcript 1; PANDA: p21-associated ncRNA DNA damage-activated; MANCR: mitosis-related lncRNA; SNHG5: small nucleolar RNA host gene 5; HOTAIR: HOX antisense intergenic RNA; UCA1: urothelial carcinoma-associated 1; NKILA: NF-κB interacting lncRNA; LNIK-A: long intergenic non-coding RNA for kinase activation; SMAD5-AS1: SMAD5 antisense RNA 1; TRIM52-AS1: TRIM52 antisense RNA 1; CACNA1G-AS1: CACNA1G antisense RNA 1; TRIM52: tripartite motif containing 52; NAALADL2-AS2: NAALADL2 antisense RNA 2; XIST: X-inactive specific transcript; SNHG16: small nucleolar RNA host gene 16

Supplementary table. Excluded studies after full-text review.

Author, Year	Title	Reason of Exclusion
**Broséus, 2016 (1)**	Relapsed diffuse large B-cell lymphoma present different genomic profiles between early and late relapses	No comparison to control group
**Dong, 2021 (2)**	Lncrna luadt1 is upregulated in mantle cell lymphoma and modulates trim11 by sponging mir-24-3p to inhibit cell apoptosis	No available full-text
**Gao, 2021 (3)**	Long non-coding RNA AFAP1-AS1 promotes cell growth and inhibits apoptosis by binding to specific proteins in germinal center B-cell-like diffuse large B-cell lymphoma	No clinical sample
**Gao, 2020 (4)**	Transcriptome profiling reveals an integrated mRNA-lncRNA signature with predictive value for long-term survival in diffuse large B-cell lymphoma	Samples from datasets
**Kang, 2020 (5)**	Systematic Analysis of Competing Endogenous RNA Networks in Diffuse Large B-Cell Lymphoma and Hodgkin's Lymphoma	Samples from datasets
**Li, 2022 (6)**	lncNBAT1/APOBEC3A is a mediator of HBX-induced chemoresistance in diffuse large B cell lymphoma cells	No clinical sample
**López, 2019 (7)**	Genomic and transcriptomic changes complement each other in the pathogenesis of sporadic Burkitt lymphoma	No related data
**Oh, 2016 (8)**	Long non-coding RNA HOTAIR expression in diffuse large B-cell lymphoma: In relation to polycomb repressive complex pathway proteins and H3K27 trimethylation	No comparison to control group
**Verma, 2015 (9)**	Transcriptome sequencing reveals thousands of novel long non-coding RNAs in B cell lymphoma	Samples from datasets
**Wu, 2022 (10)**	Regulation mechanism and pathogenic role of lncRNA plasmacytoma variant translocation 1 (PVT1) in human diseases	Review article
**Yang, 2020 (11)**	Long noncoding RNA PVT1 promotes tumor growth and predicts poor prognosis in patients with diffuse large B-cell lymphoma	Letter to editor
**Yu, 2021 (12)**	LncRNA SNHG8 Promotes Proliferation and Inhibits Apoptosis of Diffuse Large B-Cell Lymphoma via Sponging miR-335-5p	No clinical sample
**Xu, 2021 (13)**	PAX5-activated lncRNA ARRDC1-AS1 accelerates the autophagy and progression of DLBCL through sponging miR-2355-5p to regulate ATG5	Samples from datasets

**Zhang, 2022 (14)**	Epigenetic Silencing of Tumor Suppressor lncRNA NKILA: Implication on NF-κB Signaling in Non-Hodgkin's Lymphoma	No comparison to control group

References:

1. Broséus J, Chen G, Hergalant S, Ramstein G, Mounier N, Guéant JL, et al. Relapsed diffuse large B-cell lymphoma present different genomic profiles between early and late relapses. Oncotarget. 2016;7(51):83987-4002.
2. Dong L, Zhang H, Zan T, Han J, Xue Q, Sun Y. Lncrna luadt1 is upregulated in mantle cell lymphoma and modulates trim11 by sponging mir-24-3p to inhibit cell apoptosis. Critical Reviews in Eukaryotic Gene Expression. 2021;31(5):33-40.
3. Gao H, Sun Y, Chen J, Jin H, Yang W. Long non-coding RNA AFAP1-AS1 promotes cell growth and inhibits apoptosis by binding to specific proteins in germinal center B-cell-like diffuse large B-cell lymphoma. American Journal of Translational Research. 2021;12(12):8225-46.
4. Gao Q, Li Z, Meng L, Ma J, Xi Y, Wang T. Transcriptome profiling reveals an integrated mRNA-lncRNA signature with predictive value for long-term survival in diffuse large B-cell lymphoma. Aging. 2020;12(22):23275-95.
5. Kang J, Yao P, Tang Q, Wang Y, Zhou Y, Huang J. Systematic Analysis of Competing Endogenous RNA Networks in Diffuse Large B-Cell Lymphoma and Hodgkin's Lymphoma. Frontiers in Genetics. 2020;11.
6. Li J, Chen Y, Guo X, Bai X, Xu X, Han T, et al. lncNBAT1/APOBEC3A is a mediator of HBX-induced chemoresistance in diffuse large B cell lymphoma cells. Molecular Therapy - Nucleic Acids. 2022;27:1064-77.
7. López C, Kleinheinz K, Aukema SM, Rohde M, Bernhart SH, Hübschmann D, et al. Genomic and transcriptomic changes complement each other in the pathogenesis of sporadic Burkitt lymphoma. Nature Communications. 2019;10(1).
8. Oh EJ, Kim SH, Yang WI, Ko YH, Yoon SO. Long non-coding RNA HOTAIR expression in diffuse large B-cell lymphoma: In relation to polycomb repressive complex pathway proteins and H3K27 trimethylation. Journal of Pathology and Translational Medicine. 2016;50(5):369-76.
9. Verma A, Jiang Y, Du W, Fairchild L, Melnick A, Elemento O. Transcriptome sequencing reveals thousands of novel long non-coding RNAs in B cell lymphoma. Genome Medicine. 2015;7(1).
10. Wu F, Zhu Y, Zhou C, Gui W, Li H, Lin X. Regulation mechanism and pathogenic role of lncRNA plasmacytoma variant translocation 1 (PVT1) in human diseases. Genes and Diseases. 2022.
11. Yang R, Shao T, Long M, Shi Y, Liu Q, Yang L, et al. Long noncoding RNA PVT1 promotes tumor growth and predicts poor prognosis in patients with diffuse large B-cell lymphoma. Cancer Communications. 2020;40(10):551-5.
12. Yu B, Wang B, Wu Z, Wu C, Ling J, Gao X, et al. LncRNA SNHG8 Promotes Proliferation and Inhibits Apoptosis of Diffuse Large B-Cell Lymphoma via Sponging miR-335-5p. Front Oncol. 2021;11:650287.

13. Xu H, Yu X, Yang Z, Song Q, Cheng S, He Z, et al. PAX5-activated lncRNA ARRDC1-AS1 accelerates the autophagy and progression of DLBCL through sponging miR-2355-5p to regulate ATG5. Life Sciences. 2021;286.
14. Zhang MY, Calin G, Deng MD, Au-Yeung RKH, Wang LQ, Chim CS. Epigenetic Silencing of Tumor Suppressor lncRNA NKILA: Implication on NF-κB Signaling in Non-Hodgkin's Lymphoma. Genes. 2022;13(1).
